# Supplementary material for: The influence of task outcome on implicit motor learning
Source: eLife. 2019 Apr 29;8:e39882. doi: 10.7554/eLife.39882 (PMC6488295; doi:10.7554/eLife.39882)
Supplement: Supplementary file 1. [file elife-39882-supp1.docx]

**Target Size Experiment Instructions**

**Pre-Experiment Instructions (verbal):**

- “The point of this experiment is to better understand how the brain controls reaching movements. We are especially interested in how motor control is impacted by various neurological disorders, like Parkinson’s disease and cerebellar degeneration. As a healthy student, your data will be used as a normative baseline for future comparisons with these neurological patients. The information we gain from this experiment could help improve rehabilitation for these patients, so please try your best to pay attention and follow all instructions.”
- “You will be holding this ‘pen’ at the red base and should maintain the same grip throughout the experiment. (Demonstrate how to hold pen) You will be making fast center-out reaching movements towards a blue target that will appear in one of several different locations.” (Physically demonstrate the reaching movements)
- “Try your best to reach quickly and accurately in a straight line, and try to slice through the blue targets rather than stopping at the target. So, slice and come back to the home position.”
- “These are center-out reaches, which means that every trial starts from the exact same home location.”
- “During the experiment, you will see three different scenarios: you either won’t be able to see the cursor, your cursor will reflect your hand position, or you won’t be controlling the cursor at all. Regardless of what phase of the experiment you are in, your goal should always be to bring your hand directly to the blue target. And, of course, I will always tell you ahead of time which condition you are in.”
- “You will go through several different blocks of trials, some lasting longer than others, but there will be several breaks at different points of the experiment.”
- “I will give you time to adjust the seat height and scoot in close to the work station; you will be making many reaches towards the end of the tablet, and I want you to be able to do that without moving any parts of your body other than your arm.” (demonstrate)
- “Try to keep the same posture throughout the experiment.”
- “Rest your left hand in your lap.”
- “I will talk you through the first several trials.”
- “Do you have any questions?” (Run game code and switch seats.)
- “I’ll give you a minute to get comfortable before turning out the lights.”

***Start program, move mouse and keypad*

***Participant gets situated before turn off the light*

***Turn off lights and close door*

**No feedback baseline block:**

“In the center of the screen you can see a white circle that indicates the start position for each trial. When your hand is close to the start position, a cursor will appear indicating your actual hand position. For this first block of trials you won’t get to see the cursor when you reach to the target, but remember to still move your hand quickly to the target.”

(Explain “too slow” message after it happens. Remind them to make slicing movements.)

(After several trials): “That knocking sound you hear means it was a valid trial and that you moved far enough and fast enough. It does not mean you hit the target. There is no special sound for hitting the target.”

**Veridical feedback: *After first pause (screen: “Good job!”)***

“Great! The only difference for the next block of trials is that now you can see your cursor as you move to the target. Continue to move your hand directly towards the target.”

*Hit space bar.*

**Error clamp block:**

**Practice trials**

***(screen: “You will now get to familiarize yourself with the next condition.”)***

- “Great! During the next long block of trials you will continue to see the cursor, but its movement direction will ***not*** be under your control. We will want you to ignore the cursor as you ***continue to focus on slicing through the targets with your hand.”***
- “Before we start the next block, though, we’ll do three practice trials, just so that you will be familiar with exactly what will happen in the next part of the experiment.”
- “As a demonstration of what we mean by the cursor not being under your control, in the very next trial, after the blue target appears, move your hand quickly to the left.” (*hit space bar*)
- “Did you see how the cursor moved when you did, but it followed a fixed path independent of where your hand moved?”
- “I want you to now move quickly to the right after you find home and the target appears.” (*hit space bar*)
- “For the last practice trial, move in the opposite direction of the target.”

**Perturbation**

***(screen: “Ignore the cursor and move your hand directly to the target location.”)***

- “Is it clear now that the cursor’s direction will not be under your control?”
- “Those practice trials were so that you could see and experience what’s going to happen in the next block. The cursor will move like that towards all the targets now. You’ll be controlling the distance the cursor travels, but not its direction. We want to see how well you can reach to the target while ignoring the cursor.”
- “Your job is again to always reach directly for the target. But, remember, for the next block of trials you do not have control over the cursor’s direction, so try to ignore the cursor and always aim directly for the targets.”
- “Is this clear?”

After first couple of trials “Remember that you are controlling how far that cursor moves, but not its direction, even though it may look like it. No matter where you move your hand, the cursor will still travel along the same path relative to the target. Continue to ignore the cursor and move your hand directly to the target.”

*If slow, and trying to gauge cursor direction:* “Please make quick, straight reaches to the target.”

***Short break – occurs after X trials (screen: “Ignore the cursor and move your hand directly to the target location.”)***

- “Feel free to take a quick break if you’d like, but please return back to the same grip and posture when you’re ready. If you’re okay, we can just keep going. Like before, you still won’t be controlling the cursor, but we still want you to move your hand directly to the blue target.”

**No feedback washout:**

- “Great. Keep bringing your hand directly to the target. The only change is you won’t see the cursor.”

**Veridical feedback washout:**

- “Nice job. You are back in control of the cursor now. Keep bringing your hand to the target. Remember to make quick, straight reaches. Once you begin your reach, follow through with it.” (You are making sure they are not trying to make online feedback corrections)
